# Institutions for the Insane, in Prussia, Austria, and Germany

**Published:** 1854-09

**Authors:** 


*Institutions for the Iusane, in Prussia, Austria, and Germany.* By PLINY EARLE, M. D. New York: S. S. and W. Wood. 1854.

This is, in effect, a book of travels among the Asylums of Europe, recording the observations of the author in an extended tour through all the prominent institutions of the countries visited. It is only by such a comparison that a true idea of what is good and perfect can be reached. The ideas which govern the construction, superintendence, and management of insane hospitals in Europe, seem to be identical with those which obtain here in our own best institutions. As a result of a hasty examination of our author's visits, we do not find that America is at all behind Europe in enlarged ideas and generous regard for the interests of this unfortunate class, so far as these ideas have found development in the erection of asylums. In our state institutions the construction and ventilation of the houses, and the treatment of the inmates, is in accordance with the demands of science, and the dictates of humanity.

But when we turn from these to our county poorhouses, and witness there the poor manacled wretches stretched upon the hard floors of narrow cells, subjected to the control of men whose ignorance deprives them of all means of treatment save brute force and corporeal punishment, we feel that there is much reason to blush for the government which permits such cruelty. Who ever heard of a case of insanity recovering in a county poorhouse? How can they recover, when their treatment is such as would drive a sane man mad by destroying all confidence in humanity? Only one idea governs all these Bedlams—to chain and manacle the frantic, and to allow the peaceable to sink quietly into idiocy, without an attempt at cure.

Those who have read Miss Dix's account of her philanthropic visits to the alms-houses of the United States, can tell how many thousands there are confined in this hopeless manner. Those who are incredulous should make one visit to their county poorhouse. H.

